# Insights into the Role of Estrogen Receptor β in Triple-Negative Breast Cancer

**DOI:** 10.3390/cancers12061477

**Published:** 2020-06-05

**Authors:** Assunta Sellitto, Ylenia D’Agostino, Elena Alexandrova, Jessica Lamberti, Giovanni Pecoraro, Domenico Memoli, Domenico Rocco, Elena Coviello, Giorgio Giurato, Giovanni Nassa, Roberta Tarallo, Alessandro Weisz, Francesca Rizzo

**Affiliations:** 1 Laboratory of Molecular Medicine and Genomics, Department of Medicine, Surgery and Dentistry ‘Scuola Medica Salernitana’, University of Salerno, 84081 Baronissi, Italy; assellitto@unisa.it (A.S.); ydagostino@unisa.it (Y.D.); ealexandrova@unisa.it (E.A.); jlamberti@unisa.it (J.L.); gipecoraro@unisa.it (G.P.); dmemoli@unisa.it (D.M.); drocco@unisa.it (D.R.); ggiurato@unisa.it (G.G.); gnassa@unisa.it (G.N.); rtarallo@unisa.it (R.T.); 2 Genomix4Life, via S. Allende 43/L, 84081 Baronissi (SA), Italy; ecoviello@unisa.it; 3 CRGS (Genome Research Center for Health), University of Salerno Campus of Medicine, 84081 Baronissi (SA), Italy

**Keywords:** estrogen receptor β, TNBC, cancer cell metabolism, oncosuppressor

## Abstract

Estrogen receptors (ERα and ERβ) are ligand-activated transcription factors that play different roles in gene regulation and show both overlapping and specific tissue distribution patterns. ERβ, contrary to the oncogenic ERα, has been shown to act as an oncosuppressor in several instances. However, while the tumor-promoting actions of ERα are well-known, the exact role of ERβ in carcinogenesis and tumor progression is not yet fully understood. Indeed, to date, highly variable and even opposite effects have been ascribed to ERβ in cancer, including for example both proliferative and growth-inhibitory actions. Recently ERβ has been proposed as a potential target for cancer therapy, since it is expressed in a variety of breast cancers (BCs), including triple-negative ones (TNBCs). Because of the dependence of TNBCs on active cellular signaling, numerous studies have attempted to unravel the mechanism(s) behind ERβ-regulated gene expression programs but the scenario has not been fully revealed. We comprehensively reviewed the current state of knowledge concerning ERβ role in TNBC biology, focusing on the different signaling pathways and cellular processes regulated by this transcription factor, as they could be useful in identifying new diagnostic and therapeutic approaches for TNBC.

## 1. Introduction

Estrogen receptors (ERs) are ligand-activated transcription factors that mediate the effect of estrogens in the development and growth of both normal and malignant mammary tissues. Ligand-activated ERs are able to form dimers that, directly or through other proteins, bind specific estrogen response elements (EREs) in the target gene promoters and regulate their transcription [1,2].

ER alpha (ERα) is the major driver of ~75% of breast cancers (BC), its role altogether with the ones of its target genes have been extensively studied. ERα and ERα-regulated genes represent the main targets in clinical approaches aimed to control hormonally responsive BC [3].

The discovery of estrogen receptor-β (ERβ) dates back to 1996 when this molecule was cloned for the first time from rat prostate [4]. ERβ has functions and expression patterns distinct from ERα and it is widely expressed in both normal and neoplastic human breast tissues [5,6]. In the normal mammary gland, ERβ is the most widely expressed ER, and is present in luminal epithelial cells, myoepithelial cells, and some stromal cells [5]. However, studies of ERβ knockout (KO) in mice suggest a minor role of ERβ on the mammary gland development [7,8].

The exact role of ERβ in BC is controversial. Indeed, both proliferative and anti-proliferative ERβ roles have been described [9,10]. This different behavior could be explained by the existence of different receptor isoforms, problems related to the use of poorly specific antibodies (Section 3.1 and Section 3.2 of this review), and co-expression of ERβ binding partners and the use of different experimental approaches, such as ectopic expression and gene knockdown. Concerning this point, CRISPR/Cas genome editing technology could be a valid alternative to avoid artefacts or incidental effects.

Several coherent findings showed that ERβ expression decreases in precancerous and cancerous breast lesions [6,11,12,13,14,15], and is reduced or completely absent in invasive breast tumors [13,16], an event common also to other cancers such as colon, lung, ovary, and prostate [17,18]. Interestingly, Zhoa et al. [19] demonstrated an inverse correlation between methylation of ERβ promoter and mRNA levels, suggesting that ERβ expression is regulated by an epigenetic mechanism. Furthermore, in a very recent work, Warner et al. have reevaluated the role of ERβ, using the CRISPR/Cas9 technology to create an ERβ KO mouse model in which the entire gene has been deleted. These authors observed the formation of in situ ductal cancer in the prostate and mammary gland in ERβ KO mice, and then confirming an oncosuppressive role of this receptor [20].

Differences observed in the role of ERβ are also potentially related to whether or not ERβ is expressed alone or co-expressed with ERα. In fact, ERβ appears to oppose ERα actions on cell proliferation by modulating the expression of many ERα regulated genes [21], including microRNA genes [21,22]. Several research groups have explored the role of ERβ in ERα negative patient cohorts and demonstrated that ERβ expression is also associated with increased response to tamoxifen therapy [23,24].

Several reviews have generally addressed the topic of ERβ function in tumorigenesis [25,26,27,28,29], also taking into account the possible bilateral role of ERβ in BC [10]. Various reports have shown that ERβ is expressed in a subgroup of triple negative breast cancer (TNBC), accounting for 15–30% of the tissue samples (see Section 3.3) [30,31,32,33]. Characterization of the nature and role of ERβ in TNBC is little explored, but the available data suggest that its role may be different from that observed in the presence of ERα [34]. In this review, we summarize what has been learned about the role of ERβ in TNBC by highlighting the pathways regulated by ERβ that could represent a valuable target for TNBC therapies.

## 2. Pathological Features of TNBC

BC is one of the most common women cancers and represents a serious worldwide health problem. It can be considered as a multifaceted disease including a heterogeneous group of tumors with great variety in clinical, morphological, and molecular aspects [35]. Indeed, BC comprises various subtypes that differ from each other in histopathological characteristics, biomarker profiles, predictive, and prognostic parameters [36]. Traditionally, the classification of BC has been based on histological assessment and clinical staging in order to guide patient management. However, in recent years, remarkable progress in molecular analysis has profoundly improved our knowledge about BC biology and, hence, refined the classification, too [35,37]. The major classification of BC is generally based on the expression of ERα, progesterone receptor (PR) or over-expression and/or amplification of the human epidermal growth factor receptor 2 (HER2/neu); their presence is assessed to predict prognosis as well as the potential response to endocrine treatments [38,39]. Nevertheless, there is a unique subset of tumors, that accounts for almost 15–20% of all BC, characterized by the lack of expression of all three previously indicated receptor types when assessed by immunohistochemistry (IHC) and for this reason referred to as TNBC [40].

TNBCs are known to possess a more aggressive behavior and worse disease-specific outcomes compared to ERα-positive BCs. Although TNBC accounts for ~15% of all BC cases, it is responsible for over 50% of the observed mortality [41,42]. Indeed, almost one-third of the patients with TNBC face a distant recurrence within the first 3–5 years after diagnosis [43,44]. At diagnosis, patients usually present clinically positive axillary lymph node and metastatic spread to the lung, liver, and central nervous system, as well as poorer survival [45]. Given the aggressiveness of TNBC, early and accurate diagnosis of the disease is necessary for determining prognosis and allowing adequate therapy choice. Since patients affected by TNBC do not benefit from hormonal or trastuzumab-based therapies because of the lack of target receptors, to date the mainly used therapeutic approach has been surgery and cytotoxic chemotherapy, used either individually or in combination [46,47].

However, the advent of next-generation sequencing (NGS) has allowed for the dissection of TNBC biology and highlighted a number of molecular features typical of this cancer that could provide further insight into altered cellular pathways and identify novel therapeutic targets to face the disease [48,49]. Indeed, a better understanding of cancer biology together with morphological analyses can help the identification of different TNBC subtypes with various prognoses that can possibly be used to predict treatments response.

## 3. ERβ Structure and Roles in TNBC

### 3.1. ERβ Domains and Isoforms

**Figure 1:**
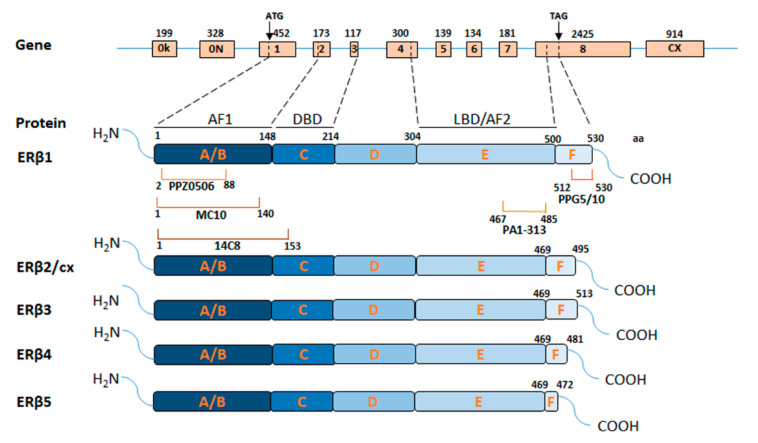
Schematic representation of ERβ gene, protein isoforms (ERβ1–5), and most used antibody epitopes. For the gene, 0K and 0N represent two promoters at the 5′ end of the gene, exons 1–8 are represented by boxes, and the introns are represented by lines. CX represents a 3′ non-coding exon present in the long form of ERβ2 protein (ERβcx). Size (bp) of each exon is showed by numbers above boxes, arrows indicate the start (ATG) and the stop (TAG) codons, and dotted lines link gene regions with the encoded protein domains. For protein isoforms, from N-terminus to C-terminus, A/B: activation function 1 (AF1) domain, C: DNA-binding domain (DBD), D: hinge domain, E: ligand-binding domain (LBD) or activation function 2 (AF2) domain, F: C-terminal domain. Square brackets show regions targeted by antibodies PPZ0506, MC10, 14C8, PPG5/10, and PA1-313. Numbers indicate the amino acids of the protein.

The full-length ERβ (also termed ERβ1), encoded by the *ESR2* gene, consists of eight exons and codify for a protein of 530 amino acids, which is structurally similar to ERα and contains five distinct domains (Figure 1) [50]. The N-terminal A/B domain, alternatively known as activation function 1 (AF1) domain, is involved in ligand-independent receptor activity and comprises several amino acids that are targeted by post-translational modifications. It shares low homology with the corresponding ERα domain and is essential for the receptor to interact with its co-regulators [51]. The C domain shares 95% identity with ERα, contains two zinc finger structures, and mediates the receptor dimerization and sequence-specific DNA binding [52]. The D domain promotes receptor nuclear translocation and is targeted by post-translational modifications that can influence ERβ activity and degradation [51]. The E domain, alternatively known as the ligand-binding domain (LBD) or activation function 2 (AF2) domain, shares 55% similarity with ERα [53] and, compared to it, has a significantly smaller ligand-binding pocket that differs in the amino acid residues lining the cavity borders, which contributes to selective receptor ligands affinity [53], opening the possibility of drug therapy with ERβ selective modulators [54]. Finally, at the ERβ C-terminal end there is a short F domain whose function is still unclear and has almost no sequence homology with ERα [52].

Besides, multiple ERβ isoforms (Figure 1) have been described and their differential expression has been shown in BC at both RNA and the protein level [55]. Beyond the full-length ERβ1, other four ERβ splicing isoforms (ERβ2/cx, ERβ3, ERβ4, and ERβ5) exist (Figure 1). These are derived mainly from alternatively splicing events involving the exon 8, resulting in C-terminally truncated proteins that cannot bind ligands but are biologically active [56].

In BC, apart from ERβ1, the best-studied isoform is ERβ2/cx, which mediates proteasome-dependent degradation of ERα [57] and its expression has been correlated with aggressive features and malignant phenotype [27,58]. ERβ2/cx and ERβ4–5 isoforms can dimerize with ERβ1, modulating its ligand-dependent transcriptional activity [59], whereas ERβ3 expression, to date, has not been detected in cell lines and tumor specimens [60], but it seems to be expressed only in testis [61].

In TNBC patients, high levels of ERβ2/cx have been associated with early tumor relapse [62]. Similar behavior of this isoform has been observed also in TNBC cell lines, where ERβ2/cx, altogether with ERβ4–5 isoforms, enhances hypoxic signaling, previously correlated to tumor aggressiveness [63]. The ERβ4 isoform, that is not expressed in physiological conditions [63], has been correlated with poor outcome in TNBC patients [64]. These evidences suggest that ERβ isoforms have distinct involvement in tumor development, which partially explain some contradictory results concerning ERβ role. However, their functions are not fully understood and further clarification is needed.

### 3.2. Issues Raised by Available ERβ Antibodies

Another explanation of the ambiguous data concerning ERβ role is the poor specificity of commercially available antibodies [65] and the lack of standardization of IHC protocols and tissue samples preparation [66]. Recently, several studies, dedicated to validation of many commonly used anti-ERβ antibodies, were published [65,66,67,68]. The obtained results are summarized in Table 1.

Antibody tests, done with different methods, indicate the best performance for three of them. The monoclonal antibody PPZ0506, which targets the N-terminal region and recognizes all 5 ERβ isoforms (Figure 1), displayed the highest receptor specificity. Its use has been validated by Western blot (WB), IHC, and immunoprecipitation coupled to mass spectrometry (IP-MS) using BC cells expressing the exogenous receptor [67]. The polyclonal PA1-313 antibody, targeting the terminal region of E domain (Figure 1), showed specific binding to ERβ1 isoform by WB and IHC in mouse xenograft tissues altogether with the ability to immunoprecipitate the receptor [32,66]. Finally, Nelson et al. reported that the monoclonal antibody MC10, which recognizes N-terminal ERβ part, is suitable for WB and specifically binds ERβ peptides in rapid immunoprecipitation mass spectrometry of endogenous proteins (RIME) assay [68]. This antibody has been also validated by Wu et al. who also demonstrated its receptor specificity by immunofluorescence and IHC analyses of BC cells and tissues [65].

The results concerning the frequently used monoclonal antibody 14C8, instead, are controversial; Andersson et al. [67], showed that it is unable to recognize the receptor in IHC experiments on paraffin-embedded ERβ-expressing cell lines; whereas Nelson et al. demonstrated its specificity in WB and its capability to immunoprecipitate ERβ in RIME, although with low specificity [68]. Discordant evidence has also been described for another commonly used antibody, the monoclonal PPG5/10, which recognizes only ERβ1 isoform; a study has reported its reliability in IHC analysis in BC cells and tissues [65], others have shown its incapability to specifically detect the receptor by WB [67,68]. The discrepancies in results obtained for PPG5/10 and 14C8 antibodies may be caused, not only by the specificity of the antibodies but also by the differences in experimental conditions used, as it was recently suggested by Gustafsson et al. [21]. Hopefully, the data summarized here will help researchers avoid the use of poorly specific antibodies and lead to a lesser discrepancy of the obtained results, to better understand the role of ERβ and its variants in cancer progression and, finally, to correctly use ERβ as a biomarker.

### 3.3. ERβ Ligands and Their Role in TNBC

ERβ, similarly to ERα, can modulate gene expression in a ligand-independent manner [69] or upon binding to its natural ligand (17β-estradiol), but also interact with several synthetic agonists and antagonists [51]. However, because of the high similarity between the ligand-binding cavities of ERα and ERβ [70], the identification of selective ligands for ERβ is still challenging, especially for the treatment of ERα-positive BC.

To date, the prevalent treatment strategies of hormone-sensitive tumors are based on the use of ERα-targeting drugs. Based on the mechanisms of action exerted, the ER ligands can be grouped into two main categories [71]: the ones that compete with estrogen and selectively modulate ERs activity (SERMs) and the ones that destabilize the ER via binding to it, inducing its degradation (SERDs). The SERMs, depending on the context where they act, can work as either agonists or antagonists. For example, tamoxifen, the most commonly used SERM in the treatment of ERα-positive BC, is known to have an anti-proliferative (or antagonistic) effect on breast tissue [72]. ERβ has been shown to bind tamoxifen [73], but the results of its action in ERα-negative BC are conflicting. Indeed, Esslimani-Sahla et al. associated low levels of ERβ with tamoxifen resistance [74] while, Hopp and colleagues demonstrated that ERβ expression had a beneficial effect on the overall survival (OS) in tamoxifen-treated tumors [75]. Finally, Barkhem and colleagues clarified that tamoxifen has an agonist/antagonist function for ERα but a pure antagonist effect for ERβ [76].

As regards SERDs, ICI 164,384 and ICI 182,780, also known as fulvestrant, are the most diffuse non-selective anti-estrogens [77], proposed as an alternative to endocrine therapy to overcome tamoxifen resistance. However, despite the well-established fulvestrant action on ERα, its role on ERβ remains controversial [78]. In TNBC, fulvestrant synergizing with tamoxifen exerts a therapeutic effect by up-regulation of ERβ [79].

Recently, most of the efforts have been focused on the identification of new selective ERβ agonists and many synthetic and natural molecules proved to be highly effective for BC prevention and treatment [80].

One of the first synthetic ERβ selective ligands reported to have a high affinity for ERβ is the 2,3-bis(4-hydroyphenyl)-propionitrile, better known as DPN. Different studies reported the use of DPN as ERβ agonist in TNBC, however, the final effects of its application are discordant; Austin et al. reported that DPN is able to activate ERβ and increase TNBC cell migration [33]; on the contrary, Song and colleagues demonstrated that ERβ activation by DPN has an inhibitory effect on migration in different BC cell lines [81]. Interestingly, ERβ-selective antagonist, ERB-041, is able to reduce proliferation in two TNBC cell lines [82].

More recently, several synthetic ligands such as LY700307, WAY200070, and 8β-VE2 have shown a high selectivity for ERβ in different experimental models and promising effects on proliferation, inhibition, and invasiveness suppression in BC, including TNBC [82,83,84,85,86], and are currently being investigated for their therapeutic use.

Among the ERβ agonists, the phytoestrogens provide a unique therapeutic opportunity to target ERβ [87]. Two of them, Liquiritigenin [88] and Genistein [89], are able to form stable complexes with ERβ, to recruit selective co-activators and interact with chromatin regulatory elements present in estrogen-responsive genes [87]. It has been reported that both these ligands act as protective factors against BC. In particular, they are able to reduce TNBC invasiveness and growth through the modulation of signaling pathways [82,90,91,92].

Finally, S-Equol, a soy isoflavone metabolite, displayed a higher ERβ selectivity when compared to the other plant-derived ligands [93]. Interestingly, it has been approved for clinical trials that started in April 2015 and aimed to determine if S-Equol is effective in decreasing the proliferation rate of TNBC (ClinicalTrials.gov Identifier: NCT02352025).

Interestingly, it has been also reported that ERβ can inhibit the pro-proliferative role of ERα also via heterodimerization with this ER subtype. Coriano et al. [94] using a multistep screening strategy (cell-based assays and in silico modeling), identified a group of heterodimer-inducing ligands that could represent a valid alternative for hormone-dependent BC targeting.

### 3.4. ERβ Prognostic Significance in TNBC

Generally, the expression levels of ERβ decrease during breast carcinogenesis [6,11,15]; from the highest expression in normal tissues [5], to the complete absence in advanced tumors [13,16]. In BC, several reports have defined ERβ mRNA as a good prognostic marker [95], whereas, others have associated ERβ presence to endocrine resistance and poor prognosis [6,96]. However, it is known that there are many problems linked with mRNA measurements in tumors since, frequently, the mRNA level does not fully predict the protein level. Evaluation of ERβ expression, based on immunostaining using validated antibodies, has confirmed the applicability of ERβ as a marker for good prognosis and prolonged disease-free survival, especially in response to tamoxifen therapy [75,97]. Data collected in ERα negative BC have also provided confounding results: some studies have shown a positive effect of ERβ1 presence, describing a reduced BC growth and invasiveness [23], and associating its high expression to good tamoxifen responsiveness [54] and to increased disease-free survival (DFS) or OS [98]; other studies indicate the negative effects of ERβ1 [30,99]; furthermore, there are studies claiming that ERβ1 presence has no prognostic significance (noteworthy, some of these results have been obtained with non-validated antibodies) [64,100,101]. Patients with ERα- and PR-negative BCs, but expressing ERβ1, instead showed a better prognosis regardless of whether the tumor is positive or not to HER2 [24].

Regarding TNBCs, Honma et al. have evaluated the clinical importance of ERβ in a cohort of 50 patients with TNBCs by IHC and associated ERβ1 positivity with significantly higher DFS and OS rate at 5 and 10 years [24]. Moreover, Wang et al. investigated ERβ1 in larger retrospective series of 571 patients with invasive TNBC, detecting the protein in ~30% of tumor samples, and demonstrated that ERβ1 presence predicts a better OS, DFS, as well as the risk factors for distant metastasis-free survival (DMFS) [102]. They also demonstrated that ERβ1 can potentially interact with the PTEN/PI3K/pAKT pathway and that ERβ1(+)/pAKT(−) status can predict the most favorable prognosis for TNBC. An opposite behavior, instead, has been described for ERβ5 isoform, whose expression in TNBC has been associated with a worse outcome [64].

## 4. ERβ Mediated Signaling Pathways in TNBC

### 4.1. ERβ Effect on Proliferation and Cell Cycle Progression of TNBC

Uncontrolled cell proliferation is a crucial step in carcinogenesis. Genetic mutations and/or epigenetic modifications can lead to abnormal activation of signaling pathways that promote cell growth and inhibit apoptosis, resulting in cell cycle deregulation [103].

In ERα positive BCs, previous studies have suggested that ERβ could exert its oncosuppressive role targeting cell division. Indeed, in breast cell lines and tumors, its expression has been inversely correlated with that of genes promoting cell cycle [104,105], whereas its exogenous expression induces inhibition of cell proliferation and tumor growth in both cell and mouse xenograft models [106].

Interestingly, anti-proliferative effects of ERβ have also been reported in ERα negative BCs, including TNBC. In the TNBC cell line MDA-MB-468, Shanle et al. reported that ERβ1 exogenous expression inhibits cell growth, arresting cell cycle at G1 phase, blocks cell colony formation and reduces tumor size in mice xenografts [107]. Furthermore, treatments with 17β-estradiol (E2) or ERB-041 enhance these effects, showing an ERβ ligand-dependent activity too. This effect is due to ERβ action on target genes, which include the ones involved in Wnt/β-catenin pathway (DKK1, WNT4, and CDH1) and in G1/S cell cycle checkpoint control (CDKN1A), two signaling pathways well-known for their role in cancer cell proliferation [108,109].

The ligand-mediated anti-proliferative effects of ERβ1 have been also demonstrated by Reese et al. in other two TNBC cell lines, MDA-MB-231 and Hs578T [110], where ectopic ERβ1 inhibits cell proliferation rate after treatments with E2 or ERβ-specific agonists, such as DPN, WAY200070, FERb 033, and Liquiritigenin. On the contrary, treatments with anti-estrogens ICI 182780, (Z)-4-hydroxy-tamoxifen (4HT), and Endoxifen restored cell growth in both cell lines. In a following study, Reese et al. demonstrated, in MDA-MB-231 cells, that ligand-mediated activation of ERβ with E2 or LY500307 impacts cell proliferation without inducing apoptosis. Cell cycle analysis, instead, revealed a G1 phase arrest, resulting from the ERβ-mediated downregulation of genes involved in cell cycle progression, including some cyclin-dependent kinases (CDK), such as CDK1 and CDK7, and the cyclins B and H [111].

Recently, Alexandrova et al. [32] have also confirmed these results in three TNBC cell lines (HCC1806, MDA-MB-468, and Hs578T), showing that exogenous expression of ERβ1 reduces cell proliferation rate increasing G1 cell cycle phase, and inhibits cell clonogenic potential of TNBC cells.

A work from 2011 [112], has also reported that induction of ERβ expression in MDA-MB-231, is able to abrogate the S-phase, and the Chk1/Cdc25C-mediated G2/M checkpoints in response to DNA damage induced by chemotherapy. ERβ seems to regulate this signaling, after the treatment with cisplatin and doxorubicin, in p53-defective BC cells but not in wild-type p53-expressing mammary cells, resulting in mitotic catastrophe and decreased cancer cell survival. These promising results suggest that ERβ presence, in cancers with defective p53, may have a predictive value for a positive response to chemotherapy.

Taken together, these evidences suggest that ERβ exerts an oncosuppressive role in TNBC by targeting cell cycle genes and slowing down the cell proliferation rate.

### 4.2. ERβ Effect on Invasiveness of TNBC

TNBCs are notoriously aggressive and prone to metastasize [44,46], but there are evidences that patients with ERβ positive tumors have shown better OS, DFS, and DMFS [24,102]. These anticancer effects seem to be related to the inhibition of canonical signaling pathways leading to blockade of metastatic phenotypes.

Different preclinical studies have demonstrated that ERβ selective targeting with agonists could be an alternative therapeutic strategy to treat TNBC [113]. For example, as reported by Zhao and colleagues, the use of LY500307 in two different mouse models is able to activate a strong antitumor innate immune response mediated by neutrophils inside metastatic niches. The study revealed that the pharmacological activation of ERβ induces IL-1β release in tumor cells and enhances innate immunity via recruitment of antitumor neutrophils, resulting in the suppression of metastasis [85]. 

**Figure 2:**
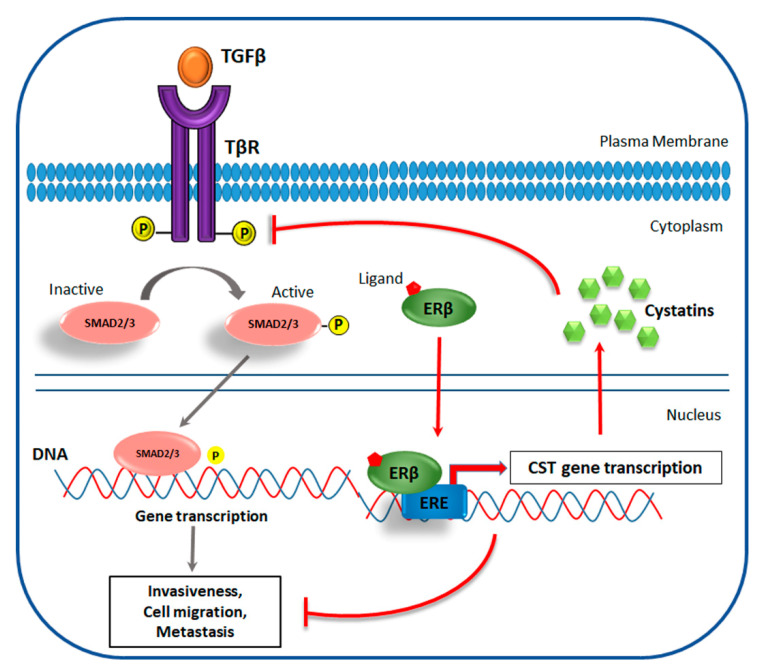
Proposed mechanism of ERβ-mediated inhibition of metastatic phenotype via suppression of TGF-β signaling in TNBC. In cancer cells, TGF-β/SMAD pathway drives invasiveness, cell migration, and metastasis formation. Ligand-activated ERβ blocks these processes by binding EREs in the CST genes, enhancing cystatin gene expression; cystatins, in turn, block canonical TGFβ signaling directly interacting with the TGFβ receptor (TβR), reducing SMAD2 and SMAD3 phosphorylation.

Reese and colleagues, using ChIP-Seq experiments and microarray analysis, identified a gene group that is differentially regulated by ERβ after E2 or LY500307 treatment. The authors demonstrated that, in TNBC, the ligand-induced activation of ERβ generates a potent anticancer effect by the inhibition of TGF-β/SMAD pathway, which normally promotes the processes of invasiveness, cell migration, and metastasis formation (Figure 2).

Indeed ERβ, through the recognition of specific ERE induces the synthesis of a secreted protein family, known as cystatins, that directly blocks TGFβ signaling and suppresses metastatic phenotypes, both in vitro and in vivo [83].

Additional molecular signaling pathways seem to be involved in the invasiveness of TNBC and the development of metastasis. It has been demonstrated that almost 80% of TNBC harbor mutations in p53 gene, a well-known oncosuppressor that regulates cell cycle and, hence, inhibits tumor proliferation. The majority of these mutations generally affect the DNA-binding domain of p53 proteins that, as a consequence, lose the tumor suppressor activity and acquire a new oncogenic function. Starting from this observation, Bado et al. demonstrated that the inhibition of p53 mutant proteins is one of the mechanisms employed by ERβ to block epithelial to mesenchymal transition (EMT) and to reduce cell-invasion in TNBC [114].

**Figure 3:**
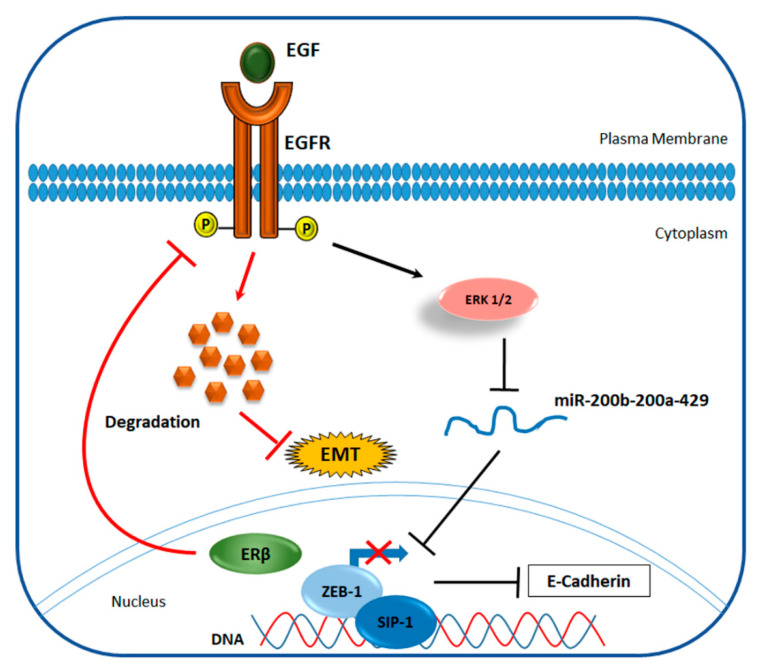
Proposed mechanism of ERβ-mediated inhibition of EMT via EGFR degradation in TNBC. EGF (epidermal growth factor), through the interaction with its receptor (EGFR), promotes epithelial to mesenchymal transition (EMT) in BC cells. Generally, EGFR signaling leads to the phosphorylation and activation of down-stream factors, such as ERK1/2 that, in turn, down-regulates the miR-200b-200a-429. This miRNA family is known to target and inhibit the action of ZEB-1 and SIP-1- transcription factors that repress E-cadherin expression. E-cadherins regulate cellular adhesion and are generally lost in EMT. ERβ blocks this network through induction of EGFR degradation, leading to up-regulation of E-cadherin protein expression and consequent EMT repression.

Moreover, a recent work reported that ERβ could inhibit EMT also by destabilizing the epidermal growth factor receptor (EGF-R), an oncogene expressed in basal-like cancers. These findings indicate that ERβ increases the expression of E-cadherins, a group of adhesion molecules that prevent cancer cell migration, via up-regulation of miR-200a/200b/429 and the consequent repression of ZEB1 and SIP1 (Figure 3), two transcription factors that generally inhibit E-cadherin synthesis [115]. Finally, a functional relationship between ERβ and the androgen receptor (AR) has also been observed. Indeed, Song et al. demonstrated that the activation of AR increases the anti-metastatic effect of ERβ by functioning as a transcription factor that directly binds to ERβ promoter [116].

Altogether, these findings suggest new promising strategies to reduce the risk of metastasis in TNBC treatment, which involve the targeting of ERβ. Interestingly, starting from May 2019, the use of E2 in treating TNBC patients ERβ positive has been approved for clinical trials (ClinicalTrials.gov Identifier: NCT03941730).

### 4.3. ERβ Effect on the Unfolded Protein Response in TNBC

Accumulation of unfolded proteins in endoplasmic reticulum (EnR), a cellular condition known as EnR stress, is observed in many cancers in response to the limited capability of tumor microenvironment to satisfy the growing request for nutrients and oxygen by cancer cells [117]. To restore protein-folding homeostasis, tumor cells activate an adaptive mechanism called unfolded protein response (UPR) [118], which provides cancer cells with alternative survival possibilities or induces cell death by apoptosis in case of severe cell damage [119]. Correlation between EnR stress signaling and cancer development was first suggested in 2004 [120] and has since been widely accepted by the scientific community [121]. In BC cells, it was demonstrated that UPR signaling promotes a malignant phenotype and can confer therapeutic resistance [122,123]. Indeed, its activation has been associated with reduced sensitivity to antiestrogens [124,125] and chemotherapy resistance [126,127].

**Figure 4:**
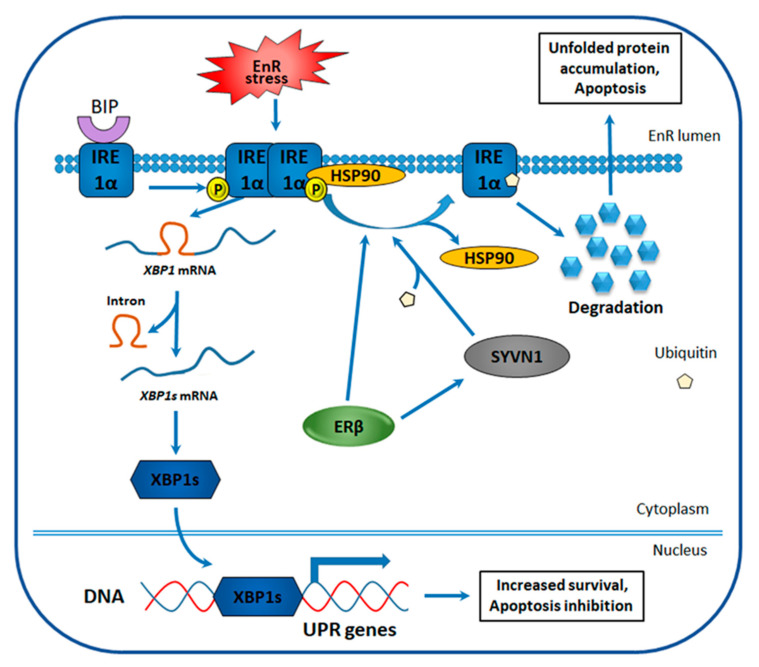
Proposed mechanism of ERβ-mediated regulation of unfolded protein response (UPR) in TNBC. Endoplasmic reticulum (EnR) stress activates inositol-requiring enzyme 1α (IRE1α), IRE1α self-dimerizes and undergoes autophosphorylation, then IRE1α induces X-box-binding protein 1 (XBP1) mRNA splicing with the formation of spliced XBP1 (XBP1s) mRNA. XBP1s-encoded protein functions as a potent transcription factor that triggers UPR-involved gene expression, whose expression promotes cell survival and inhibits apoptosis. ERβ induces dissociation of heat shock protein 90 (HSP90) from IRE1α and increases the expression of Synoviolin 1 (SYVN1) that ubiquitinates IRE1α. Both processes are known to induce IRE1α degradation, leading to downregulation of pro-survival XBP1s, unfolded protein accumulation in EnR, and apoptosis.

One of UPR signaling branches is regulated by inositol-requiring enzyme 1α (IRE1α), an EnR transmembrane protein that contains functional kinase and RNase domains. In normal conditions, IRE1α is bound to binding immunoglobulin protein (BIP) that prevents its dimerization and dissociates in response to EnR stress (Figure 4) [128]. When released from BIP, IRE1α self-dimerizes and undergoes autophosphorylation leading to a conformational change that activates the RNase domain. Activated IRE1α then splices a 26-nucleotide intron from the mRNA encoding for X-box-binding protein 1 (XBP1), causing an open reading frame shift and expression of an activated XBP1 form–spliced XBP1 (XBP1s) [129,130]. XBP1s functions as a transcription factor and induces the expression of genes involved in protein folding, EnR-associated protein degradation and protein secretion, whose activation abolishes the accumulated unfolded protein [131,132].

There are several evidences indicating that IRE1α-XBP1 UPR signaling branch plays a role in TNBC tumorigenicity, one of which is the correlation between high XBP1s expression level and poor prognosis observed in TNBC patients [133]. Another study showed that XBP1 depletion in TNBC cell line models inhibits tumor growth, decreases tumor relapse and the CD44^high^CD24^low^ population of cancer stem cells [134], known to mediate cancer recurrence [135,136,137]. Analysis of XBP1 chromatin binding demonstrated that XBP1 cooperates with another transcription factor hyper-activated in TNBC, the hypoxia-inducing factor 1α (HIF1α) [138,139], which recruits RNA polymerase II transcriptional machinery and drives the expression of HIF1α target genes [134].

ERβ involvement in the TNBC cell sensibilization to EnR stress-stimulating therapy was suggested basing on the enhanced apoptosis observed in ERβ1-expressing TNBC cells treated with these drugs [140]. Investigation of underlying mechanisms revealed that although unspliced XBP1 expression is not influenced by ERβ, XBP1s amount is significantly reduced in the presence of the receptor in EnR stress conditions [140]. Corroborating this observation, the mRNA level of XBP1s target genes, known to participate in UPR, was also decreased. The same experiments were also performed for another ERβ isoform–ERβ2, whose upregulation failed to induce apoptosis and inhibit XBP1s expression in response to EnR stress, indicating the specificity of the observed effects for full-length ERβ. Further research revealed that the inhibition of XBP1s expression is a result of reduced IRE1α protein expression in ERβ-positive cells [140]. At the same time, IRE1α mRNA expression, in the absence of EnR-induced stress, was not affected, indicating an ERβ-mediated post-transcriptional regulation of IRE1α expression, known to occur through increased ubiquitination and degradation. Two proteins are known to regulate IRE1α degradation: E3 ubiquitin ligase, Synoviolin (SYVN1), and the molecular chaperone heat shock protein 90 (HSP90). SYVN1 ubiquitinates IRE1α, whereas HSP90 binds IRE1α to protect it from degradation and renders IRE1α accessible to proteases upon dissociation from it [141,142]. Evaluation of their possible role in TNBC revealed that ERβ-expressing cells overexpress SYVN1 and are characterized by a decreased association of IRE1α with HSP90 (Figure 4) [140]. These results indicate that ERβ presence sensitizes TNBC cells to EnR stress-inducing drugs through repression of IRE1α-XBP1 signaling branch of UPR.

### 4.4. ERβ Effect on the Bioenergetics of TNBC

Energy metabolism represents a fundamental molecular process in cancer cells and a better understanding of mechanisms, underlying its regulation, can provide new therapeutic strategies to selectively eliminate cancer cells by targeting their unique metabolism.

Adenosine triphosphate (ATP) production in normal cells relies primarily on a process called oxidative phosphorylation (OXPHOS), that occurs in the mitochondria and is regulated by mitochondrial DNA (mtDNA)-encoded genes [143]. This way of ATP production is highly efficient and is preferred by most body cells. Cancer cells, instead, even in aerobic conditions favor ATP production by glycolysis, a phenomenon known as the Warburg effect in oncology [144]. Aerobic glycolysis occurs in the cytoplasm and is characterized by enhanced enzymatic activity and increased glucose uptake by cancer cells. Among the main factors causing this metabolic shift there are aberrant oncogene activity, loss of tumor suppressor expression, hypoxic microenvironment, mutations in mtDNA, and impairment of mitochondrial function [145,146,147].

Analysis of metabolic phenotype of TNBC tissues revealed that, similarly to genomic heterogeneity, TNBCs are characterized by high metabolic heterogeneity [148,149]. There is some evidence indicating the correlation between the metabolic characteristics of TNBCs and therapeutic responsiveness. For example, it was shown that exposure of TNBC cells to high glucose concentrations induces proliferation and abrogates the apoptotic effects induced by the drug metformin [150]. Other studies demonstrated that combined treatments, inducing the metabolic switch from glycolysis to OXPHOS, increases the sensitivity of TNBC cells to mitochondrial respiratory complex I inhibitors [151]; whereas inhibition of aerobic glycolysis enhances antitumor efficacy of Zoptarelin Doxorubicin, a cytotoxic agonist of gonadotropin-releasing hormone receptor (GnRH-R), expressed in more than 70% of TNBC tumors [152]. Altogether, these studies indicate that combinational treatment, including energy metabolism-targeting agents, may represent a therapeutic strategy for aggressive TNBC treatment.

**Figure 5:**
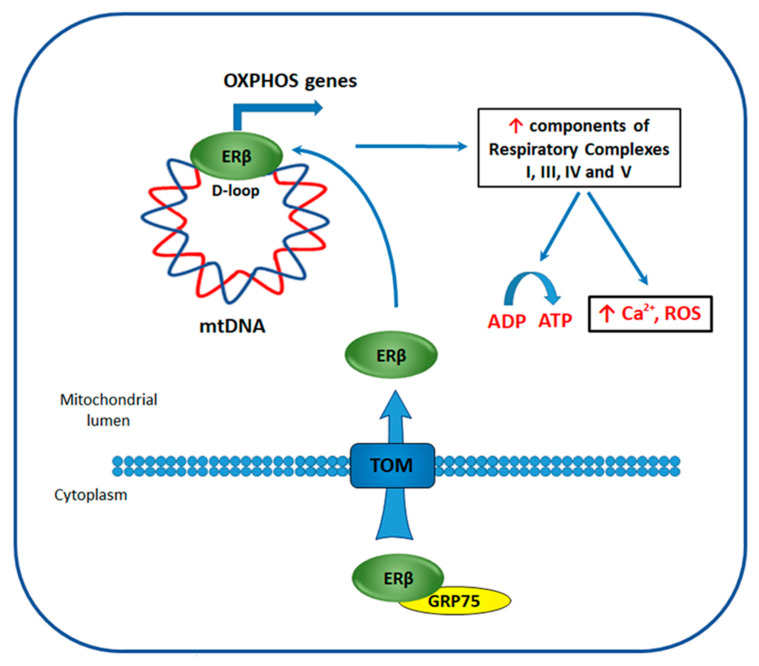
Proposed mechanism of ERβ-mediated regulation of oxidative phosphorylation (OXPHOS) in TNBC. ERβ interacts with glucose-regulated protein 75 (GRP75) and undergoes translocation into the mitochondria with the aid of the translocase of the outer membrane (TOM) complex. In mitochondria, ERβ binds to mitochondrial DNA (mtDNA) in displacement loop (D-loop) region and drives the expression of genes encoding for the components of respiratory complexes I, III, IV, and V, responsible for OXPHOS. OXPHOS activation leads to an increase of mitochondrial Ca^2+^, reactive oxygen species (ROS), and ATP concentrations.

ERβ localization in mitochondria has been described for the first time by Yang et al. [153] in 2004, who observed the presence of ERβ-mapping peptides in human heart mitochondria lysate by mass spectrometry (MS) [153]. This result suggested that estrogens could directly affect the functions of this important organelle through ERβ, hypothesis further confirmed by numerous studies. Indeed, ERβ was detected in mitochondria of rat (uterus, ovarian, hippocampus, and neuronal cells) and human (cardiomyocytes, sperm, periodontal ligament, and cultured lens epithelial cells) tissues, altogether with BC (MCF7), hepatocarcinoma (HepG2), and osteosarcoma (SaOS-2) cell lines [154]. It was suggested that transportation of ERβ into the mitochondria occurs through the targeting of TOM20 and/or TOM70 import receptors, both belonging to the translocase of the outer membrane of mitochondria (TOM) complex (Figure 5), but through different mechanisms [155]. For example, TOM 20 functions as preprotein receptor and preferentially binds to presequences ϕXXϕϕ (where ϕ and X are hydrophobic and any amino acids respectively), it imports ERβ into the mitochondria through interaction with one of the four ERβ presequence motifs. TOM 70, instead, may transport ERβ with the aid of molecular chaperone complex, formed by HSP70 and HSP90 heat shock proteins, by docking through tetraticopeptide repeat motifs (TPR) [155]. Indeed, in MCF7 cells it was demonstrated that ERβ translocation to mitochondria is dependent on receptor interaction with glucose-regulated protein 75 (GRP75), a chaperone protein belonging to Hsp70 family [156].

Mechanisms of mitochondrial ERβ action has been extensively studied in hormone-responsive BC, where it was demonstrated that treatment with E2 enhances ERβ mitochondrial localization in a concentration- and time-dependent manner [157]. Moreover, it was shown that ERβ, through the binding to EREs present in the mtDNA displacement loop (D-loop) (Figure 5) [158], regulates the expression of mtDNA genes encoding for proteins of the mitochondrial respiratory chain, such as cytochrome c oxidase subunit (CO) I, CO II, CO III, ATP synthase subunits 6 and 8, and the mitochondrial transcript precursor [159].

Moreover, the specific evaluation of ERβ mitochondrial role in TNBC cells has demonstrated that its mitochondria-targeted (mitoERβ) expression is able to inhibit proliferation, cell cycle progression, and reduce colony and spheroid formation [156]. These effects were abrogated upon deletion of C- or N-terminal fragments of mitoERβ protein in cell culture and mice xenograft models [156]. Further analysis revealed that full-length mitoERβ expression induces transcription of 13 mitochondrial genes through binding to mtDNA D-loop, an effect that was not observed in case of the introduction of C- or N-terminally-truncated receptor variants. Among 13 genes, whose expression was affected, components of respiratory complexes I, III, IV, and V were present, including mtDNA-encoded genes regulated by ERβ in hormone-responsive BC. Finally, increased levels of mitochondrial Ca^2+^, reactive oxygen, and ATP production were observed in the presence of mitoERβ [156]. In our previous study [32], GRP75 was present among putative ERβ interactors, determined by MS, in TNBC cells indicating its possible role in the receptor translocation to mitochondria also in this BC subtype. All these results indicate that ERβ activates transcription of mtDNA-encoded genes leading to the activation of OXPHOS in TNBC cells.

### 4.5. ERβ Effect on Cholesterol Biosynthesis

Cholesterol biosynthesis is another metabolic process, whose reprogramming favors cancer cells adaptation to environmental conditions and sustain an increased proliferation rate [160,161]. Cholesterol is critical for cellular homeostasis maintenance and necessary for membrane integrity and fluidity, being a key component of the plasmatic membrane lipid rafts that scaffold extracellular signaling [162]. Moreover, cholesterol represents an essential precursor for the synthesis of steroid hormones (e.g., estrogen and progesterone [163]), vitamins (e.g., vitamin D), and bile acids. Cellular cholesterol comes from two main sources: external environment and intracellular synthesis. Cancer cells are often characterized by high cholesterol level, which is fueled more by increased intracellular synthesis than by serum cholesterol [161,164,165], suggesting that altered cholesterol homeostasis may trigger or at least support tumorigenesis.

Deregulation of cholesterol biosynthesis occurs by multiple processes including cholesterol import, synthesis, export, metabolism, and esterification [161,162]. Sterol regulatory element-binding proteins (SREBPs) are the main regulators of these metabolic processes, and function as transcription factors that bind sterol regulatory elements and drive expression of more than 30 lipid biogenesis genes [166]. Three SREBP proteins, SREBP1a, SREBP1c, and SREBP2, are known. They differ by their preferences in gene expression activation. SREBP1a is a potent driver of expression of all SREBP-responsive genes, including the ones necessary for cholesterol, fatty acids, and triglycerides biosynthesis; whereas SREBP1c and SREBP2 actions are more specific and comprise transcription of fatty acid synthesis for SREBP1c and cholesterol biosynthesis genes for SREBP2 [167]. Cholesterol biosynthesis in cancer is known to be regulated by PI3K/AKT/mTOR, RTK/RAS, and mutated p53 through SREBP activation by mechanisms that have been previously reviewed [160,161].

Analysis of cholesterol metabolic pathway activity in BC revealed that TNBC tumors are characterized by a hyper-activated cholesterol biosynthesis and efflux compared to the majority of ERα-positive BC tumors [168,169]. Recently, in TNBC cells, it was described that cholesterol metabolism is regulated by RAR-related orphan receptor gamma (RORγ), which facilitates SREBP2 recruitment to cholesterol biosynthesis genes and whose genetic and pharmaceutical inhibition causes tumor regression, thus representing an excellent target for TNBC treatment [168]. Other important regulators of cholesterol metabolism in TNBC are p53 mutants, derived from wild type protein by substitutions of amino acids R280K or R273H, located in p53 DNA-binding domain and directly interacting with DNA. These two p53 mutants, partly through the cooperation with SREBP, associates with sterol gene promoters leading to the activation of mevalonate pathway and causing a significant phenotypic effect on breast tissue architecture [164]. Moreover, it was demonstrated that p53 mutations correlate with high expression of genes encoding for components of cholesterol biosynthesis in BC [164] that, considering high p53 mutation rate in TNBC [170], suggests that targeting cholesterol biosynthesis could be a treatment option for patients with TNBC.

**Figure 6:**
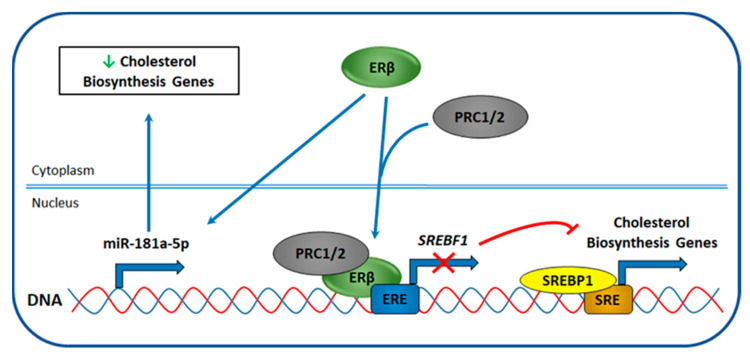
Proposed mechanism of ERβ-mediated regulation of cholesterol biosynthesis in TNBC. ERβ interacts with chromatin repressive complexes, e.g., polycomb repressive complexes 1 and 2 (PRC1/2), binds to ERE present in sterol regulatory element binding factor 1 (SREBF1) gene promoter and inhibits SREBF1 expression. SREBF1 gene encodes for sterol regulatory element binding protein 1 (SREBP1), which drives expression of cholesterol biosynthesis genes by binding to sterol regulatory elements (SREs) present in their promoters. Inhibition of SREBF1 transcription reduces expression of SREBP1-driven genes leading to the downregulation of cholesterol biosynthesis. Alternatively, ERβ by unknown mechanism induces expression of miR-181a-5p, which targets cholesterol biosynthesis genes and regulates their expression post-transcriptionally.

Recently, Alexandrova et al. [32] described that ERβ regulates cholesterol biosynthesis in TNBC through inhibition of all three branches of this metabolic pathway. This observation was based on transcriptomic analyses performed in three TNBC cell lines, belonging to different subtypes and expressing exogenous ERβ, pointing to a subtype-independent effect of this receptor in TNBC. SREBP1 and SREBP2 were among the upstream regulators found responsible for the observed phenotype and genome-wide analysis of ERβ binding to TNBC cell genome and the nature of its molecular partners revealed that ERβ inhibits SREBP1 expression by binding to its DNA regulatory elements and recruiting chromatin repressive complexes such as polycomb repressor complexes 1 and 2 (PRC1/2) (Figure 6), that probably suppress SREBP1 expression through the introduction of repressive epigenetic marks [32]. Further investigation of mechanisms of ERβ-mediated regulation of cholesterol biosynthesis revealed that small non-coding RNAs are also involved in the regulation of this metabolic process. In particular, it was demonstrated that miR-181a-5p expression could be regulated by ERβ by an unknown mechanism (Figure 6). In fact, its upregulation has been described in both TNBC cell lines, after induction of ERβ expression, and in ERβ-positive vs. -negative biopsies. Moreover, among the predicted targets of miR-181a-5p, key components of cholesterol biosynthesis were present [171]. Altogether, these results indicate the involvement of different epigenetic mechanisms in ERβ-mediated regulation of cholesterol biosynthesis in TNBC.

### 4.6. ERβ Effect on AR Signaling Pathways

Recent researches have revealed that a group of TNBC expresses the AR, allowing the definition of a luminal androgen receptor (LAR) subtype [172,173]. Moreover, it has been demonstrated that AR plays a pro-proliferative activity in preclinical models, which opened the possibility of using two anti-androgens, bicalutamide and enzalutamide, in TNBC treatment [174,175,176]. Clinical trials for both drugs in metastatic TNBC have revealed promising results and a higher clinical benefit for enzalutamide [175,176]. Moreover, in 2016 started a phase II clinical trial, aiming to assess the use of enzalutamide in early-stage AR-positive TNBC (NCT02750358). A recent study proposed a connection between ERβ and AR receptor in TNBC, suggesting that ERβ oncosuppressive role in AR-positive TNBC is mediated by the direct and indirect interactions with AR [177]. The authors, using MDA-MB 453 cells, demonstrated that the ERβ presence increases the sensitivity of TNBC cells to anti-androgens, especially to enzalutamide. In fact, enzalutamide treatment enhances the formation of AR:ERβ heterodimer, preventing AR from forming homodimers. Then the heterodimer enters the nucleus where it is unable to bind androgen-responsive element (ARE) sequences and promote the expression of cell growth genes. These results suggest that a new therapeutic option, consisting of a combined therapy targeting both receptors, may be useful for TNBC patients, whose tumors express both ERβ and AR receptors.

## 5. Conclusions

TNBC is an extremely aggressive BC subtype that lacks well-defined therapeutic targets and for this reason, has limited treatment options, which is prompting researchers to study the molecular mechanisms that drive TNBC onset and progression. One of the suggested targets for TNBC treatment is ERβ, but to date, its role in cancer progression is still debated because of several reasons, the main of which are poor specificity of available ERβ- recognizing antibodies and existence of several receptor isoforms [55] that exert an opposite effect on the cancer growth (discussed in Section 3.1). Recently, a strong effort has been made to solve some of these problems and several works were published where the performance of the most frequently used anti-ERβ antibodies has been validated using different techniques [65,66,67,68]. The obtained results are summarized in Section 3.2 and Table 1. Using extensively validated anti-ERβ antibodies, it has been demonstrated that 15–30% of TNBCs express the ERβ receptor [32,102], but the data concerning prognostic value of the receptor expression in TNBC patients are still scarce and additional studies using larger and better-characterized patient cohorts are necessary, especially in relation to different ERβ isoforms. Nevertheless, there are multiple experimental data demonstrating the anti-proliferative and anti-migratory effects of exogenous ERβ1 in TNBC cell lines and mice xenograft models [32,107,110,111], indicating the possibility of antitumor ERβ1 action also in vivo. Importantly, the existence of selective ERβ agonists [80,84,85,87], summarized in Section 3.3, allows to enhance the tumor suppressor effect guided by the receptor in vitro, and, once the problems with ERβ detection in human tissues is solved, they could be successfully used to activate ERβ in TNBC patients, without stimulating the pro-proliferative program driven by ERα in normal human cells.

In this review, we have highlighted the ERβ involvement in the different molecular pathways composing the complex network of signaling processes, regulating TNBC onset and progression.

The most interesting mechanisms of ERβ action in TNBC are linked to the regulation of cellular metabolic programs, which takes place through ERβ-mediated downregulation of UPR in the presence of EnR stress [140], induction of OXPHOS by expression of mtDNA-encoded genes [156] and inhibition of cholesterol biosynthesis by suppression of SREBF1 gene transcription [32] or upregulation of miR-181a-5p [171]. ERβ is also able to drive expression of cystatins, that promote inhibition of TGFβ signaling leading to the reduced metastatic potential of TNBC cells in vitro and in vivo [83]. Moreover, ERβ has been associated several times with drug sensitivity. In fact, there is evidence that ERβ expression sensitizes tumor cells to anti-androgen therapy, in AR-positive TNBC model [177], and to chemotherapy drugs doxorubicin and cisplatin in the presence of defective p53 [112].

Altogether, these data indicate that TNBC patients may potentially benefit from administration of ERβ-targeted therapy singularly or in combination with chemotherapeutical drugs [178], anti-androgen therapy [179,180], EnR stress-inducing substances [181,182], or inhibitors of cholesterol biosynthesis [183,184], some of which are already used for the treatment of other diseases. There is no doubt that all these treatment strategies require further extensive validation and testing, and a better understanding of the ERβ-dependent regulatory mechanisms, revised here may be useful toward the identification of new and effective ways to treat ERβ-positive TNBCs.

## Figures and Tables

**Table 1:** Details of the main ERβ antibodies applications and performance.

Reference	ERβ Antibodies	Source	Tested Applications	Performance
Wu et al. [65]	pAb AB1410	Chemicon	IHC in human cell lines, IF	Bad
	mAb GR40	Calbiochem	IHC in human cell lines, IF	Bad
	mAb MC9	Homemade	WB, IP	Good
	mAb MC10	Homemade	WB, IP, IHC in human cell lines and tissues, IF	Good
	mAb PPG5/10	Thermo Fisher Scientific	IHC in human cell lines and tissues, IF	Good
	pAb sc-6820	Santa Cruz Biotechnology	IHC in human cell lines, IF	Bad
Shanle et al. [66]	pAb PA1-313	Thermo Fisher Scientific	WB, IHC in mouse xenograft tissues	Good
Nelson et al. [68]	mAb 14C8	Abcam	WB, RIME	Low specificity for RIME application
	mAb CWK-F12	DSHB	WB, RIME, IHC in human cell lines	Good
	mAb GeneTex 70182	GeneTex	WB, RIME	Good
	mAb MC10	provided by Wu *et al.* 2012	WB, RIME	Good
	pAb Millipore 06-629	Millipore	WB, RIME	Bad for WB application
	mAb NCL-ER-BETA	Leica Biosystems	WB, RIME	Bad
	mAb PPG5/10	Thermo Fisher Scientific	WB, RIME	Bad for WB application
	pAb Sc8974	Santa Cruz Biotechnology	WB, RIME	Good
Andersson et al. [67]	mAb 14C8	GeneTex	WB, IP, IHC in human cell lines and tissues	Bad for WB, IP and IHC in human tissues applications
	mAb PPG5/10	DAKO	WB, IP, IHC in human cell lines and tissues	Bad
	mAb PPZ0506	Invitrogen	WB, IP, IHC in human cell lines and tissues	Good
Alexandrova et al. [32]	pAb PA1-313	Thermo Fisher Scientific	IP	Good

pAb, polyclonal antibody, mAb, monoclonal antibody, IHC, immunohistochemistry, IF, immunofluorescence, WB, western blot, IP, immunoprecipitation, RIME, rapid immunoprecipitation mass spectrometry of endogenous proteins.

## Abbreviations

4HT	(Z)-4-hydroxy-tamoxifen
ATP	Adenosine Triphosphate
AF1/AF2 domain	Activation Function 1/2 domain
AKT	Protein Kinase B
AR	Androgen Receptor
ARE	Androgen-Responsive Element
BC	Breast Cancer
BIP	Binding Immunoglobulin Protein
Cas	CRISPR-associated protein
Csc25C	M-phase inducer phosphatase 3
CD24	CD24 antigen
CD44	CD44 antigen
CDH1	Cadherin-1
CDK	Cyclin-Dependent Kinase
CDKN1A	Cyclin-Dependent Kinase Inhibitor 1A
ChIP-Seq	Chromatin Immunoprecipitation Sequencing
Chk1	Checkpoint Kinase 1
CO	Cytochrome C Oxidase
CRISPR	Clustered Regularly Interspaced Short Palindromic Repeat
D-loop	mtDNA Displacement loop
DBD	DNA-Binding Domain
DFS	Disease-Free Survival
DKK1	Dickkopf-related protein 1
DMFS	Distant Metastasis-Free Survival
DPN	3-bis(4-hydroyphenyl)-propionitrile
E2	17β-estradiol
EGFR	Epidermal Growth Factor Receptor
EMT	Epithelial to Mesenchymal Transition
EnR	Endoplasmic Reticulum
ERα	Estrogen Receptor α
ERβ	Estrogen Receptor β
ERE	Estrogen Response Element
ERK1/2	Extracellular Regulated MAP Kinase1/2
GRP75	Glucose-Regulated Protein 75
HER2/neu	Human Epidermal growth factor Receptor 2
HIF1α	Hypoxia-Inducing Factor 1α
HSP90	Heat Shock Protein 90
IF	Immunofluorescence
IHC	Immunohistochemistry
IL-1β	Interleukin 1 beta
IP	Immunoprecipitation
IP-MS	Immunoprecipitation coupled to Mass Spectrometry
IRE1α	Inositol-Requiring Enzyme 1α
KO	Knockout
LAR	Luminal Androgen Receptor
LBD domain	Ligand-Binding Domain
mAB	Monoclonal Antibody
mitoERβ	Mitochondria-targeted ERβ
MS	Mass Spectrometry
mtDNA	Mitochondrial DNA
mTOR	Mammalian Target of Rapamycin
NGS	Next Generation Sequencing
OS	Overall Survival
OXPHOS	Oxidative Phosphorylation
p53	Tumor protein p53
pAB	Polyclonal Antibody
PI3K	Phosphatidylinositol 3-Kinase
PR	Progesterone Receptor
PRC1/2	Polycomb Repressor Complex 1/2
RAS	Protein belonging to small GTPases superfamily
RIME	Rapid Immunoprecipitation Mass spectrometry of Endogenous proteins
RORγ	RAR-related Orphan Receptor Gamma
ROS	Reactive Oxygen Species
RTK	Receptor Tyrosine Kinase
SMAD	Homolog of the *Caenorhabditis elegans* SMA and *Drosophila* MAD family of genes
SERD	Selective Estrogen Receptor Degrader
SERM	Selective Estrogen Receptor Modulator
SIP1	Survival of motor neuron protein-Interacting protein 1
SRE	Sterol Regulatory Element
SREBF	Sterol Regulatory Element Binding Factor
SREBP	Sterol Regulatory Element-Binding Protein
SYVN1	Synoviolin 1
TGFβ	Transforming Growth Factor beta
TβR	Receptor of TGFβ
TNBC	Triple-Negative Breast Cancer
TOM	Translocase of the Outer Membrane of mitochondria
TPR	Tetraticopeptide Repeat motifs
UPR	Unfolded Protein Response
WB	Western Blot
WNT4	Wnt family member 4
XBP1	X-box-Binding Protein 1
XBP1s	Spliced XBP1
ZEB1	Zinc finger E-box-binding homeobox 1
